# Clinical application of confocal laser endomicroscopy in neurosurgery: a scoping review

**DOI:** 10.3389/fsurg.2025.1715836

**Published:** 2026-01-28

**Authors:** Yuan Xu, Carlos E. Calderon-Valero, Thomas J. On, Francesco Restelli, Francesco Acerbi, Jürgen Schlegel, Evgenii Belykh, Mark C. Preul

**Affiliations:** 1 The Loyal and Edith Davis Neurosurgical Research Laboratory, Barrow Neurological Institute, St. Joseph’s Hospital and Medical Center, Phoenix, AZ, United States; 2 Department of Neurosurgery, Fondazione IRCCS Istituto Neurologico Carlo Besta, Milan, Italy; 3 Department of Translational Research and New Technologies in Medicine and Surgery, University of Pisa, Pisa, Italy; 4 Department of Neurosurgery, Pisa University Hospital, Pisa, Italy; 5 Department of Neuropathology, Institute of Pathology and Molecular Diagnostics, University of Augsburg, Augsburg, Germany; 6 Department of Neurosurgery, Rutgers University, Newark, NJ, United States

**Keywords:** 5-aminolevulinic acid (5-ALA), central nervous system tumor, confocal laser endomicroscopy (CLE), fluorescein sodium (FNa), fluorescence-guided surgery (FGS), indocyanine green (ICG), intraoperative pathology

## Abstract

**Background:**

Confocal laser endomicroscopy (CLE) is an emerging intraoperative “optical biopsy” tool that enables real-time, *in vivo*, cellular-resolution visualization of brain tumor histoarchitecture. It offers the potential to complement frozen section pathology by providing rapid intraoperative feedback. We conducted a scoping review of prospective clinical studies to characterize CLE platforms, fluorophores, operative applications, and diagnostic performance in neurosurgical patients.

**Methods:**

This review followed PRISMA-ScR guidelines. A systematic search of PubMed, Scopus, and Embase was performed. Eligible studies were prospective clinical studies of intraoperative CLE imaging in neurosurgical patients. Two independent reviewers screened and extracted data on study design, CLE system, fluorophore use, pathology types, diagnostic performance, and workflow characteristics.

**Results:**

From 379 initial records, 19 studies met final inclusion criteria, with most (63%) in the past five years. Five CLE platforms were studied: CONVIVO (47%), FIVE1 (16%), Cellvizio (16%), EndoMAG 1 (11%), and cCeLL (11%). These CLE systems use different fluorophores that lead to distinct image characteristics. Across tumor types, CLE demonstrated diagnostic accuracy comparable with frozen section, with reported sensitivity up to 93% and specificity up to 94% in certain scenarios. CLE interpretation was feasible within minutes, faster than frozen section, and several studies reported successful integration with fluorescence-guided surgery and telepathology platforms.

**Conclusions:**

Clinical evidence supports the feasibility, safety, and efficiency of CLE in neurosurgery, offering rapid intraoperative histology without tissue removal. Current studies remain observational with varying study design and outcome definition, limiting assessment of the effectiveness and impact. Well-designed interventional trials are needed to determine CLE's definitive role as an intraoperative optical biopsy tool guiding tumor resection and patient outcomes.

## Introduction

Confocal laser endomicroscopy (CLE) has emerged over the past decade as a powerful optical biopsy tool capable of providing real-time, *in vivo* high-resolution visualization of tissue histoarchitecture (1). Originally developed mainly for gastrointestinal and pulmonary endoscopy, CLE systems have been adapted for neurosurgical use in both *ex vivo* and *in vivo* settings. These probe-based systems employ miniaturized fiberoptic probes that emit a laser of a specific wavelength, usually in the blue or infrared range, which match the fluorophore used to create contrast between cellular structures and the background.

Traditional intraoperative frozen-section pathology remains the gold standard for intraoperative diagnosis, assessing tumor margins and guiding extent of resection. Yet, it incurs delays, requires tissue removal, and is susceptible to sampling error and interpretation variability (2). Compared to other intraoperative guidance and diagnostic methods, such as widefield fluorescence guidance, intraoperative MRI, intraoperative ultrasound, stimulated Raman histology, etc. have advanced operative decision-making but differ in resolution, workflow integration, and accessibility. CLE offers unique real-time cellular-resolution imaging without tissue removal, bridging the gap between widefield fluorescence and histopathology. It has the potential to augment or even replace frozen section pathology without interrupting workflow.

Early animal studies demonstrated that the use of the CLE probe to image brain tissue is possible (3, 4). Subsequent clinical investigations across North America, Europe, and Asia have evaluated multiple CLE systems that had distinct features and generated different images. In recent years, with several CLE systems receiving clinical clearance, the number of well-structured studies increased. Therefore, we performed a scoping review of all prospective clinical studies of intraoperative CLE imaging in neurosurgical patients to characterize the technology platforms, fluorescent contrasts, operative settings, and diagnostic outcomes reported to date. Our objectives were to analyze reported CLE diagnostic performance, delineate its potential impact on the intraoperative workflow of brain tumor surgery, and identify areas for future innovation and standardization in neurosurgical optical biopsy.

## Material and methods

### Literature search strategy

This review was conducted in accordance with the PRISMA-ScR guidelines (5). A systematic search for all publications containing reports of clinical use of intraoperative neurosurgical CLE imaging, including prospective clinical studies, case series and case reports, was conducted to identify all eligible articles. Three authors (YX, CEC, and TJO) collaborated to develop a comprehensive search strategy. Three major biomedical databases—PubMed, Scopus, and Embase—were searched without application of language or date restrictions on June 20, 2025. The search strategy included title/abstract and terms commonly used for the CLE technology. Supplemental hand searching was performed to identify additional records. The complete search strategies, including research terms, are available in Supplementary Table 1.

### Data extraction

Database search results were exported and uploaded to Rayyan, an online platform for reference screening. All references were independently reviewed by 2 review authors (CEC, TJO). Only prospective clinical studies, case series and case reports of intraoperative CLE imaging in neurosurgical patients were included. Animal studies and retrospective analysis CLE databases of previously reported studies were excluded to avoid potential inclusion of repetitive data. Discrepancies were resolved by a third author (XY) as the tiebreaker. Data extraction was done by 2 authors (XY, CEC) independently from each report, and disagreements were resolved through discussion and consensus between reviewers. The extracted data were stored in a electronic form created by the authors. Data extracted from each eligible study included 1) article information, such as authors and affiliation, country in which the study was conducted, publication year; 2) CLE system used and modality of imaging (*ex vivo* or *in vivo*); 3) fluorophore type, dosage, route and timing of administration; 4) number of cases and types of pathology; 5) diagnostic image features; 6) diagnostic performance; 7) time required to reach diagnosis.

### Synthesis of results

Due to the heterogeneity in the study design, presence of descriptive and nonquantitative reports, difference in outcome definition and reported metrics, a quantitative synthesis was not attempted. Instead, description of the individual studies, including the extracted data, important findings and conclusions were reported.

## Results

### Literature search results

**Figure 1:**
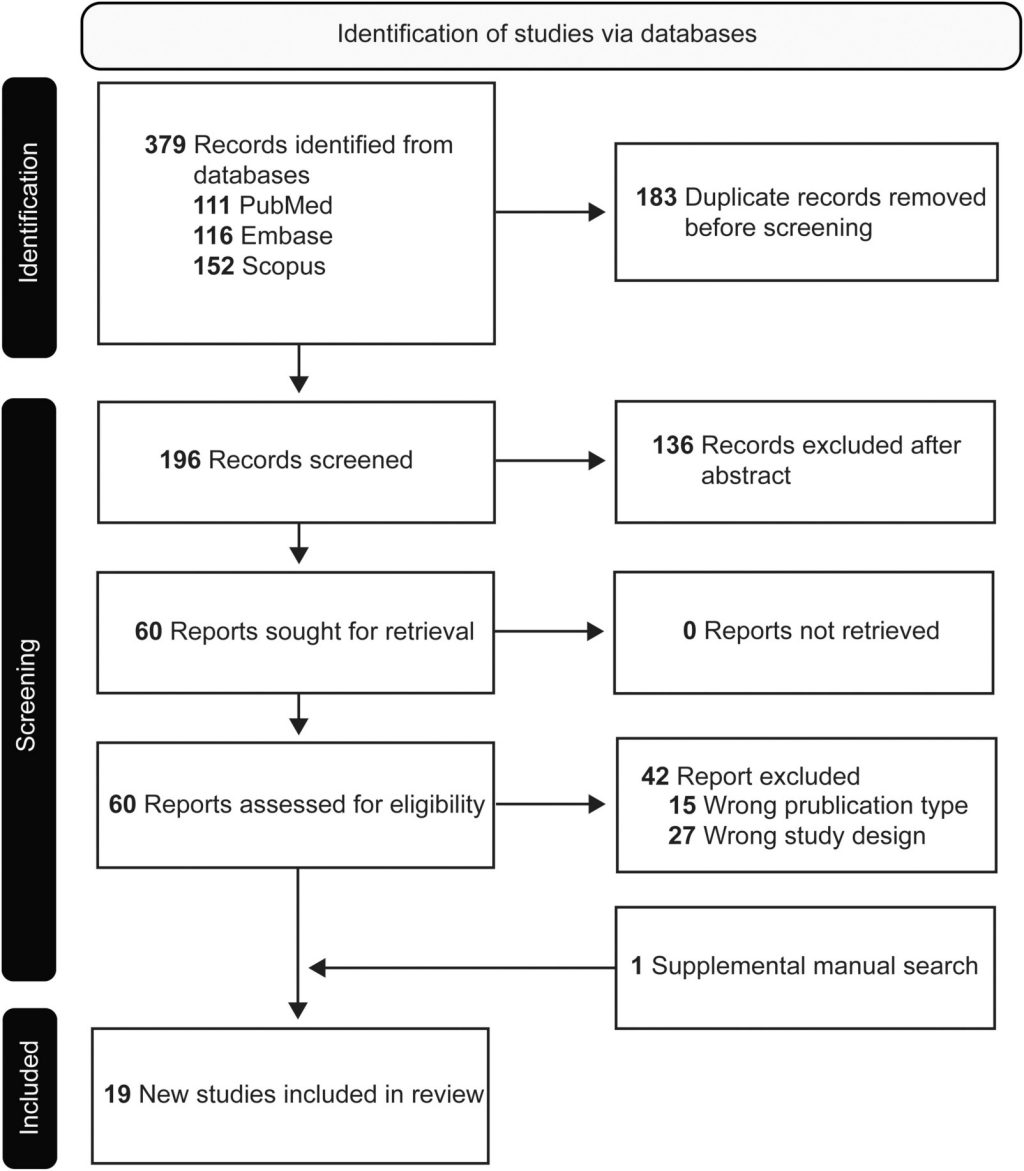
PRISMA flow diagram of systematic review of prospective clinical studies of intraoperative CLE imaging during neurosurgical procedures, outlining the database searched, number of initial records and nonduplicate records, the articles excluded, and the final 19 included reports. Used with permission from Barrow Neurological Institute, Phoenix, Arizona.

The initial search resulted in 379 references. After duplicates were removed (*n* = 183), 196 unique records were identified and included in the screening process. After initial screening of abstracts, 136 entries did not meet inclusion criteria and were removed, most often because they were animal studies only, not reporting original study data, or not using probe-based clinical CLE imaging systems. Full text retrieval was attempted for the remaining 60 reports. After full text review, 42 studies were excluded. Supplemental hand searching resulted in 1 study that were included in this review after full text review. A total of 19 articles met the final inclusion criteria (see Figure 1 for the PRISMA flow diagram).

The 19 articles were published between 2011 and 2025 (Table 1), with 12 (63%) of the articles published within the last 5 years (2020–2025). Ten studies (53%) were published by European institutes; seven (37%) were published by a single US institute; two (11%) were published by South Korean institutes. Five different CLE system paired with specific fluorophores were used across the 19 studies: FIVE1 with FNa (Optiscan Imaging Ltd., Melbourne, Australia, *n* = 3, 16%), Cellvizio with acriflavine, FNa, 5-ALA, and ICG (Mauna Kea Technologies, Paris, France, *n* = 3, 16%), EndoMAG 1 without fluorescent contrast (Karl Storz GmbH, Tuttlingen, Germany, *n* = 2, 11%), CONVIVO with FNa (Carl Zeiss Meditec AG, Jena, Germany, *n* = 9, 47%), and cCeLL with ICG (VPIX Medical, Daejeon, Republic of Korea, *n* = 2, 11%). Data extracted were summarized and presented in Table 1. Basic specifications of the five CLE imaging systems were summarized in Table 2.

**Table 1:** Extracted data from clinical reports of neurosurgical CLE imaging.

Extracted data	Studies (*N* = 19), *n* (%)
Study design	
Prospective clincal studies	18 (95)
Case report	1 (5)
Geographic distribution of institutions	
Europe	10 (53)
USA	7 (37)
South Korea	2 (11)
CLE systems	
FIVE1, Optiscan Imaging Ltd.	3 (16)
Cellvizio, Mauna Kea Technologies	3 (16)
EndoMAG 1, Karl Storz, GmbH	2 (11)
CONVIVO, Carl Zeiss Meditec AG	9 (47)
cCeLL, VPIX Medical	2 (11)
Imaging modality	
*in vivo*	8 (42)
*Ex vivo*	8 (42)
Both	3 (16)
Fluorophore used	
FNa	11 (58%)
ICG	3 (16%)
AF, topical	1 (5)
5-ALA	1 (5)
None	3 (16%)

**Table 2:** Comparison of the CLE systems evaluated for neurosurgical use.

CLE system	CONVIVO	cCeLL	Cellvizio	FIVE1	EndoMAG 1
Fluorophore	FNa	ICG	FNa, ICG, 5-ALA	FNa	None
Laser wavelength (nm)	488	775	488 or 780	488	670
Field of view (µm)	475 × 267 (rectangular, 16:9)	500 × 500 (square)	Round, diameter varies across probes	475 × 475 (square)	300 (diameter, round)
Maximum resolution	1,920 × 1,080 pixels	1,024 × 1,024 pixels	1 µm/pixel	1,080 × 1,080 pixels	Axial: 2 µm, Lateral: 1–2 µm
Scanning depth (µm)	−50 to 200	Fixed	Fixed, varies across probes	0–500	Up to 300
Scanning speed (FPS)	0.75–2.35	10	12	0.83–1.25	40

FPS, frames per second, FNa, fluorescein sodium, ICG, indocyanine green, 5-ALA, 5-aminolevulinic acid.

### FNa-based CLE systems

FNa-based CLE imaging systems were the first CLE imaging systems studied and reported in the context of neurosurgery. Two generations of fluorescein-based CLE technology have been used in neurosurgical studies, both sharing the same laser and optic components. The Gen1 preclinical CLE system, FIVE1 developed by Optiscan Imaging Ltd., is a probe-based confocal imaging system with a 488 nm wavelength blue laser and various bandpass and long pass filters for green-yellow light, compatible with the absorption-emission spectrum of the clinically approved FNa (6). The first CLE system adapted for clinical neurosurgery, the Zeiss CONVIVO *in vivo* pathology suite, harbors the Optiscan Gen2 CLE system. Compared to the Gen1 system, the Gen2 system received quality-of-life upgrades mainly in probe ergonomics, user interface and metadata handling, but saw a 44% decrease in single frame field of view (FOV) from 475 × 475 µm to 475 × 267 µm due to a transition from 1:1 screen ratio to fit a 16:9 display. A new z-stack function allows for a quick 3-dimensional evaluation of tissue (6).

The first ever reported use of CLE system in neurosurgical patients was a prospective feasibility study of a prototype FNa-based CLE system evaluated its use during 33 brain tumor surgery of gliomas, meningiomas, metastatic tumors and necrosis (7). FNa was administered intravenously at a dose of 2–5 mg/kg several minutes before the imaging starts. Using the CLE probe affixed by most surgeons to a Greenberg retractor system, the researchers performed real-time, *in vivo* cellular resolution imaging, or optical biopsies, of brain tumor tissue and in the tumor-brain interface to assess tumor histology, grade, and margins. The study qualitatively showed that CLE imaging could both visualize microvasculature and identify histological features of various tumor types. Importantly, the authors reported that both neurosurgeons and neuropathologists successfully differentiated between high- and low-grade gliomas, oligodendrogliomas and astrocytoma, and tumor margins and normal parenchyma. This preliminary feasibility study validated the utility of this novel intraoperative imaging technology in neurosurgery and paved way for future research. However, it did not utilize a clinically approved system for neurosurgery by the US Food and Drug Administration (FDA) or European CE Mark (Conformité Européenne). A follow-up study at the same institution evaluated the diagnostic potential of CLE imaging in 50 patients undergoing brain tumor resection (8). In total, 88 *in vivo* optical biopsies were done in various tumor types, including meningioma, glioma, schwannoma, ependymoma, and hemangioblastoma and compared to matched histopathological sections acquired at the same location. Results showed strong concordance between CLE findings and conventional histology. Specifically, hallmark histological features such as collagen fibers and psammoma bodies in meningiomas and fascicles of cells with elongated cytoplasmic processes in schwannomas were identified in CLE images. In-low and high-grade gliomas, the degree of tumor cellularity and atypia was consistent with matched histological sections. A neuropathologist who lacked significant prior experience with interpreting CLE images reviewed a set of 28 images with information about tumor location and enhancement and yielded a 92.9% diagnostic accuracy, comparable to the accuracy of frozen section biopsies. The authors proposed that this digital imaging system could play a significant role in tumor margin assessment and real-time telepathology.

Martirosyan et al. (9) conducted a large prospective clinical study to evaluate the sensitivity and specificity of intraoperative CLE imaging in 74 consecutive patients by comparing CLE findings to matched histological sections. In addition to analyzing the feasibility and qualitative image interpretation of CLE imaging, this paper emphasized statistical validation, methodical histopathological correlation, and workflow integration. Both *in vivo* and *ex vivo* CLE imaging was done, with imaged tissue processed for histological processing and analysis. 46% of the *in vivo* images and 53% of the *ex vivo* images were diagnostic. CLE achieved high diagnostic accuracy with sensitivity and specificity for gliomas at 91% and 94%, and for meningiomas at 97% and 93%, respectively, comparable to frozen section performance. However, this study did not include a detailed comparison between *in vivo* and *ex vivo* imaging modalities. Nevertheless, the results highlighted CLE's potential to streamline and enhance intraoperative neuropathological interrogation.

Prior to receiving USFDA clearance and EU CE Mark for *in vivo* use in 2019, CONVIVO underwent *ex vivo* trials in human patients. In the US, Belykh et al. (10) reported the results from a single-center *ex vivo* study involving 47 patients undergoing fluorescence-guided brain surgery. The study evaluated the diagnostic accuracy of CLE imaging for real-time intraoperative imaging of brain tumor microstructure. A total of 122 optical biopsies were acquired *ex vivo* immediately following surgical resection using CLE with corresponding frozen and permanent sections as the gold standard. Overall, CLE demonstrated a high positive predictive value (97% overall, 98% for gliomas) and specificity (90% overall, 94% for gliomas). Diagnostic accuracy was highest when images were interpreted by experienced neuropathologists or neurosurgeons. Distinct histological features seen in CLE images were described, with bright background, hypercellularity, atypia most representative of underlying tumor pathology. Interestingly, an unusually high 40 mg/kg dose of FNa given intravenously at induction of anesthesia to a patient with a non-enhancing, presumed low-grade glioma resulted in improved CLE visualization of tissue histoarchitecture that is uncommon with these tumors (11). In addition, CLE image quality notably improved after a second FNa injection during the latter half of the surgery, on average 2.6 h after the first dose. These findings underscore the feasibility and diagnostic potential of CLE as a portable, noninvasive optical biopsy tool, and support its integration into fluorescence-guided neurosurgical workflows and future *in vivo* applications.

In Europe, another single-center study with a similar design was conducted by Acerbi et al. (12) to assess the diagnostic performance of *ex vivo* CLE imaging in 15 newly diagnosed glioblastoma patients undergoing fluorescein-guided resection. Both tumor core and margins were sampled, imaged with CLE *ex vivo*, and processed for frozen and permanent histology. Blinded, near real-time interpretation of CLE images led to high concordance with frozen sections—80% for pathology diagnosis and 93.3% for morphology description at the tumor core. Concordance was slightly lower at the margins (66.6% for pathology diagnosis and 80% for morphology description). When compared with permanent sections, a lower concordance was also seen at tumor margins, reflecting the diagnostic challenge in infiltrative zones. CLE images reliably identified hallmark features of GBM, such as hypercellularity, necrosis, and vascular proliferation. The average time for CLE interpretation was under 6 min, supporting the feasibility of intraoperative use.

Following its approval for *in vivo* use in patients, CONVIVO underwent several *in vivo* feasibility studies. The first observational study was published by Höhne et al. (13), evaluating the feasibility, safety, and intraoperative utility of *in vivo* CLE imaging in 12 patients undergoing fluorescence-guided surgery with FNa for various central nervous system tumors. FNa was given at a dose of 5 mg/kg intravenously, and time between FNa dosing and CLE imaging varied significantly. Tissue from three distinct tumor regions, namely tumor core, tumor border, and potential infiltration zone, were imaged with CLE, and yielded representative images of different pathologies. Shorter time interval between FNa administration and CLE imaging resulted in more assessable images. Intraoperative *in vivo* CLE imaging was well tolerated with no adverse events. The authors demonstrated successful integration into the surgical workflow without complications and indicated the need for further evaluation of this intraoperative imaging modality.

Abramov et al. (14) revealed the diagnostic performance of *in vivo* CLE imaging from systematic analysis of their prospective clinical study involving 30 patients with 31 intracranial lesions (gliomas, meningiomas, metastases, and others). A standard dose of 5 mg/kg FNa was given to each patient intravenously within 5 min before CLE imaging. Interpretable images were successfully obtained in all cases. Percentage of interpretable images improved with user experience. CLE demonstrated high diagnostic performance, with 92% concordance with permanent histology. Notably, the addition of a novel telepathology software platform facilitated real-time remote neuropathology consultation by securely transferring CLE images to the neuropathologist who was not in the operating room and enabling real-time voice communication between the operating neurosurgeon and the neuropathologist (15).

**Figure 2:**
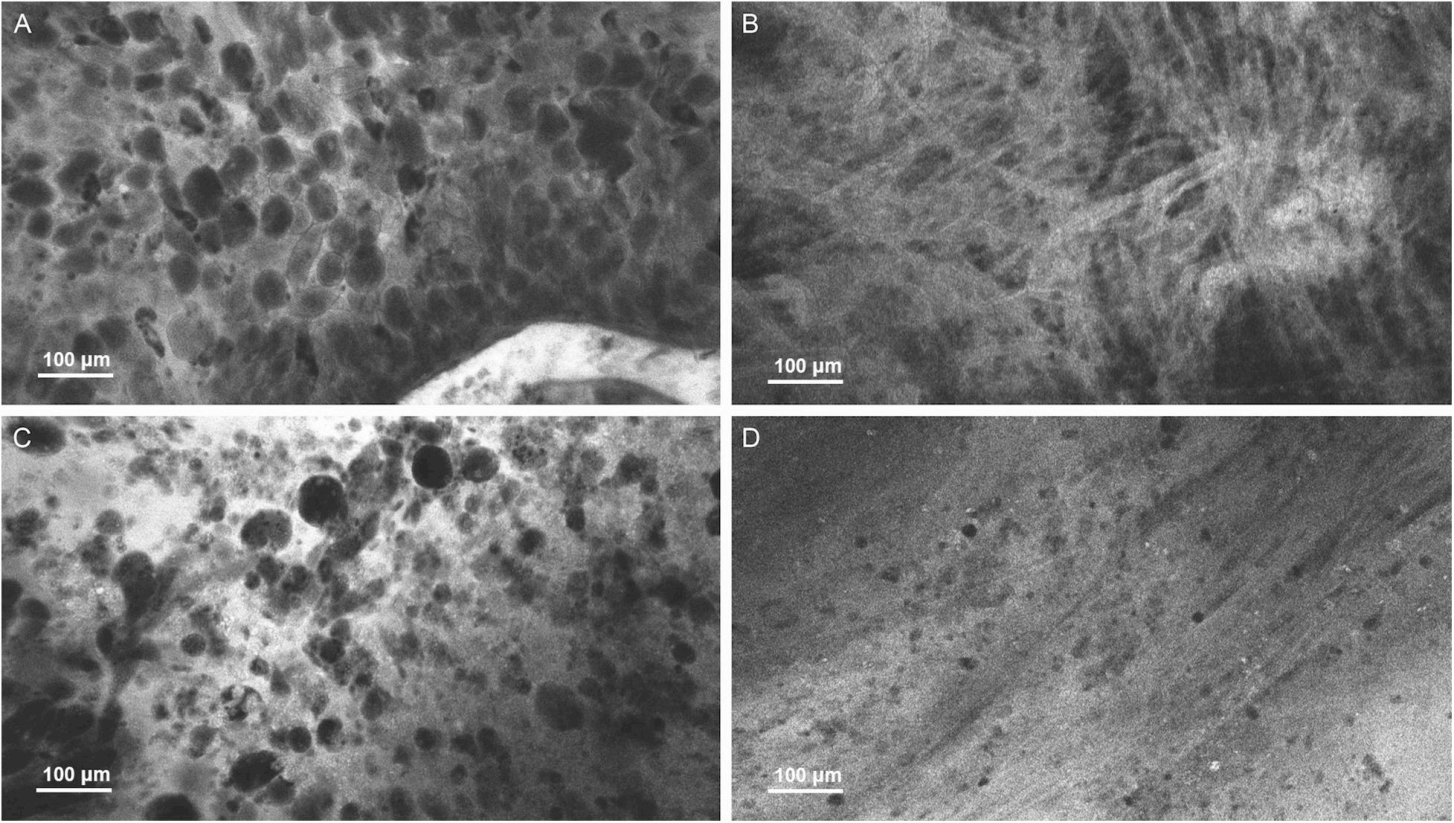
Examples of CLE images acquired *in vivo* using CONVIVO in **(A)** glioblastoma, **(B)** meningioma, **(C)** metastatic breast cancer, and **(D)** post-treatment effect. Used with permission from Barrow Neurological Institute, Phoenix, Arizona.

This study was later expanded to include an additional 20 patients, primarily with gliomas, increasing the total cohort to 50 patients (Figure 2) (1). In total, over 13,000 interpretable images were collected from 304 regions of interest (ROIs), with the first diagnostic images typically obtained within 10.5 s of imaging initiation. The CLE system achieved an overall sensitivity of 93% and specificity of 81%, comparable to the diagnostic performance of frozen section. Notably, at glioma margin ROIs, both sensitivity (85% vs. 94%) and specificity (50% vs. 93%) were lower compared to the glioma core. As a result, the positive and negative likelihood ratios at the margins were 1.7 and 0.3, respectively, indicating limited diagnostic value for confirming or excluding tumor presence in these regions. The accuracy of the FNa-based CLE system to discriminate regions of brain post-treatment effect (Figure 1D) was near 100%. Real-time telepathology consultation was used in 27 cases, enhancing intraoperative interpretation but increasing imaging time modestly by 3.8 min. A socioeconomic analysis found that in the US healthcare setting according to Medicare standards, the cost of intraoperative CLE imaging is similar to that of frozen sections and may reduce costs by up to two-thirds compared to 5-ALA fluorescence-guided brain tumor surgery. The study demonstrated that CLE is a safe, efficient, and cost-effective intraoperative guidance tool delivering real-time, high-resolution histological visualization without the need for tissue removal, offering comparable diagnostic performance to frozen section but without the delay associated with tissue processing. Its reduced specificity at tumor margins, however, required investigation for better performing fluorophores.

**Figure 3:**
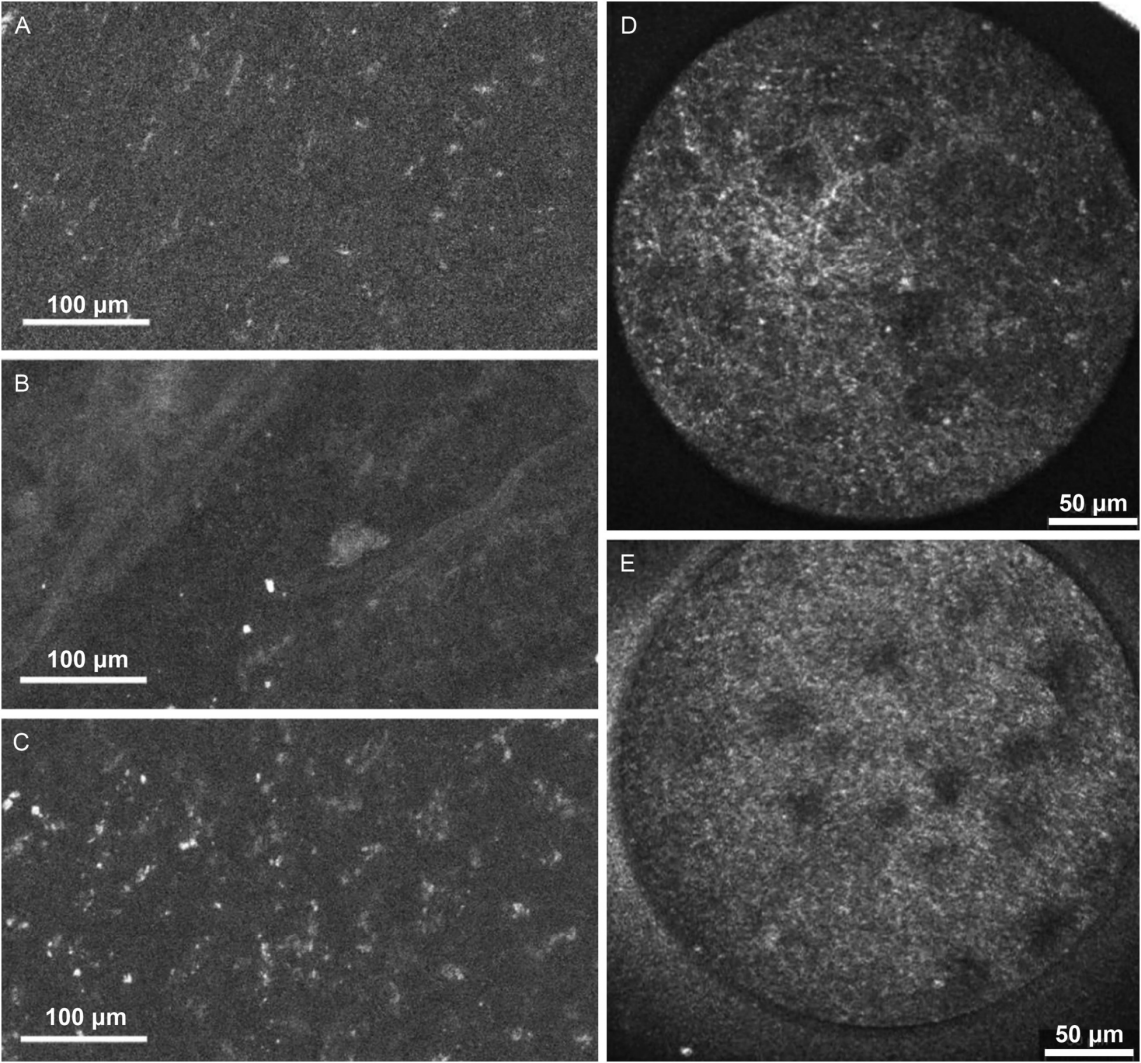
Examples of label-free CLE images acquired using CONVIVO *in vivo* in **(A)** glioblastoma, **(B)** meningioma, and **(C)** non-tumor tissue, and using EndoMAG 1 *ex vivo* in **(D)** low-grade glioma and **(E)** high-grade glioma. Panels A-C are reproduced from Reichenbach et al. (2024) (16), under the Creative Commons Attribution 4.0 International License (http://creativecommons.org/licenses/by/4.0/). Panels 3D and 3E are adapted with permission from Breuskin et al. (2017) (25), © 2017 Elsevier Inc.

Another interesting study published by Reichenbach et al. (16) on label-free CLE imaging revealed the autofluorescence pattern of different tissue types (Figures 3A–C). FNa was not given to the patient or used to treat tissue prior to CLE imaging. Tissue samples from 26 cases, including 6 glioblastomas, 6 meningiomas, 9 metastases, 2 pituitary adenomas, and 2 surgically resected epilepsy foci, were collected and imaged with CLE within 2 h of resection. *in situ* label-free CLE imaging was done in 3 cases. Label-free multiphoton microscopy and H&E-stained histology were used as references. The bandpass filter produced images with lower background intensity compared to the longpass filter, making the autofluorescence easier to see, although no different in signal-to-background ratio was found between bandpass and longpass filter images. Distinct autofluorescence features were described, including punctate autofluorescence of unknown significance, strong agglomerate cytoplasmic autofluorescence in various tumor types and nontumor tissues, and fibrous and round structures corresponding to elastin fibers and psammoma bodies in meningiomas. These observations provided unique insight into the autofluorescence phenomenon that is often obscured by the strong FNa fluorescence signal and overlooked, although the significance and association of autofluorescence patterns with tumor, nontumor pathology and normal brain tissue need to be better studied.

The multi-center clinical trial by Wagner et al. (17) is the largest prospective clinical study of FNa CLE so far. To compared diagnostic performance of CLE imaging and frozen section, 210 patients from 3 participating sites were included, encompassing a range of intracranial pathologies, including gliomas, meningiomas, and metastases. FNa was injected intravenously 20–40 min before the planned tumor resection. CLE imaging was done only once during each case on the surface of presumed tumor tissue. A histological diagnosis was given by a trained neuropathologist, and the concordance between CLE and final histopathology was calculated. Results showed that the accuracy of intraoperative CLE imaging was noninferior to standard frozen section (87% vs. 91%, *p* = 0.367). The median time needed to reach a diagnosis using CLE was 3 min and 27.5 min using frozen section. Blinded interpretation by two neuropathologists demonstrated high interrater reliability for images consistent with tumor tissue, but low agreement for nontumor samples. Despite the large cohort size, this study is limited by its design that led to a low number of digital biopsy sampling and lack of sampling in different areas around the tumor, especially relevant for comparing image interpretation in the mass of a glioma vs. margin regions. Nonetheless, this study demonstrated the relatively high accuracy and efficiency of intraoperative CLE imaging.

The latest prospective clinical study by Restelli et al. evaluated *in vivo* CLE imaging at the resection margins (18). Seventy-five patients with possible aggressive tumors in not-primary-eloquent regions of the brain undergoing fluorescein-guided surgery were enrolled. FNa was administered at the induction of anesthesia and used for widefield fluorescence guidance. Virtual CLE biopsies were obtained *in vivo* from both the tumor core and resection margins, assessed blindedly by neuropathologist, and compared with standard histology. At the margins, CLE achieved an overall accuracy of 85.8% (sensitivity 89.6%, specificity 79.4%) across all tumors, and 82.2% accuracy in glioblastoma/grade 4 IDH-mutant astrocytomas. At the tumor core, diagnostic concordance with histology was 67.6% overall and 80.9% in grade 4 gliomas, with higher agreement for morphological and cellularity features. The mean time for CLE interpretation was less than four minutes, underscoring its efficiency for intraoperative use. These findings demonstrated the capability of CLE imaging in identifying the presence of infiltrating tumor at the cellular level at the margins in real time, offering a potential adjunct to maximize the extent of resection in central nervous system tumors. Further multicenter studies and integration with artificial intelligence (AI) are warranted to confirm its role in improving surgical outcomes.

### ICG-based CLE system

ICG is another clinically approved fluorophore with both excitation (780∼805 nm) and emission (820∼840 nm) wavelengths in the near-infrared (NIR) range. Thus, the fluorescence of ICG cannot be directly visualized with naked eye. A NIR CLE system using ICG as the fluorescent contrast developed by Optiscan was initially explored in mouse glioma models in the very early stage of development, showing adequate penetration of tumor tissue by ICG and good visualization of tumor cells (3). Later, another NIR CLE system, cCeLL, was developed by VPIX Medical. This system is equipped with a 775 nm laser and an 800∼860 nm bandpass filter designed for ICG (19). A motorized auto-stage is available for *ex vivo* use of the probe to control the position of the probe for *ex vivo* imaging purposes.

**Figure 4:**
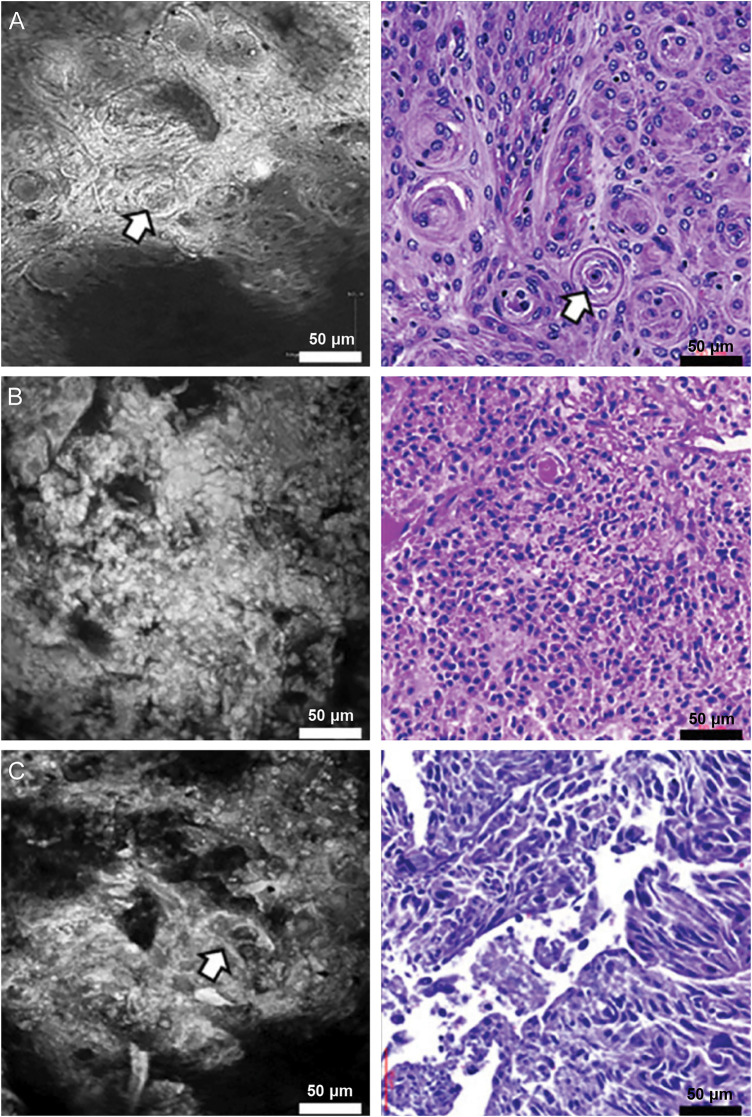
Examples of CLE images acquired *ex vivo* using cCeLL in **(A)** meningioma, **(B)** glioblastoma, and **(C)** metastatic adenocarcinoma. Adapted from Hong et al. (2023) (19), under the Creative Commons Attribution License (CC BY).

Hong et al. (19) reported the first *in vitro* experiments with human U87 and mouse GL261 glioma cell lines and *ex vivo* experiments with U87, GL261, and 2 patient-derived glioma cell lines, CSC2 and X01, showing good delineation of tumor cells that was detected with cCeLL. *Ex vivo* imaging of 3 normal brain and 69 human brain tumor specimens reproduced hallmark microstructures of each subtype. Tissues removed during surgeries (including gliomas of different WHO grades, meningiomas, pituitary adenomas, metastases, lymphomas, and others) were incubated in 0.5 mg/mL ICG solution and imaged with CLE. Pathognomonic histoarchitectures were observed with distinct tumor types, allowing for discrimination of different pathologies. (Figure 4) Interestingly, glioma grade correlated positively with cellularity and inversely with streak-like patterns consistent with axons visible on CLE. Finally, the authors demonstrated panoramic, three-dimensional reconstructions by combining Z-stack merging and image stitching to overcome the device's limited FOV.

In a follow-up multi-center prospective clinical trial, Byun et al. (20) analyzed the diagnostic efficacy of *ex vivo* imaging with cCeLL compared to frozen section for intraoperative diagnosis. Seventy-four biopsy specimens from 55 newly diagnosed brain tumor patients, with the majority (74.3%) sampled from the tumor core but also from tumor margin and normal tissue, were divided for permanent histology, frozen section, and *ex vivo* CLE imaging. All CLE images were interpreted by one neuropathologist, while frozen sections were interpreted by pathologists with specializations in various fields depending on institutional circumstances. Prior to CLE imaging, tissue samples were incubated in 2.5 mg/mL ICG solution in the dark for 5 min. All CLE imaging and frozen section were categorized as tumor or nontumor and compared to permanent histology to calculate the diagnostic performance. cCeLL-*ex vivo* achieved a relatively high overall accuracy of 89.2%, slightly higher than frozen section (86.5%). Both *ex vivo* CLE imaging and frozen section yielded a sensitivity of 92.2%, while CLE imaging had higher specificity (70%) than frozen section (50%). Moreover, CLE reduced time from tissue preparation to diagnosis by more than half (13 min 17 s vs. 28 min 28 s, *p* < 0.005). These results suggest that rapid, high-resolution ICG-based CLE could serve as a supplementary tool for intraoperative brain tumor diagnosis.

### Other CLE systems

**Figure 5:**
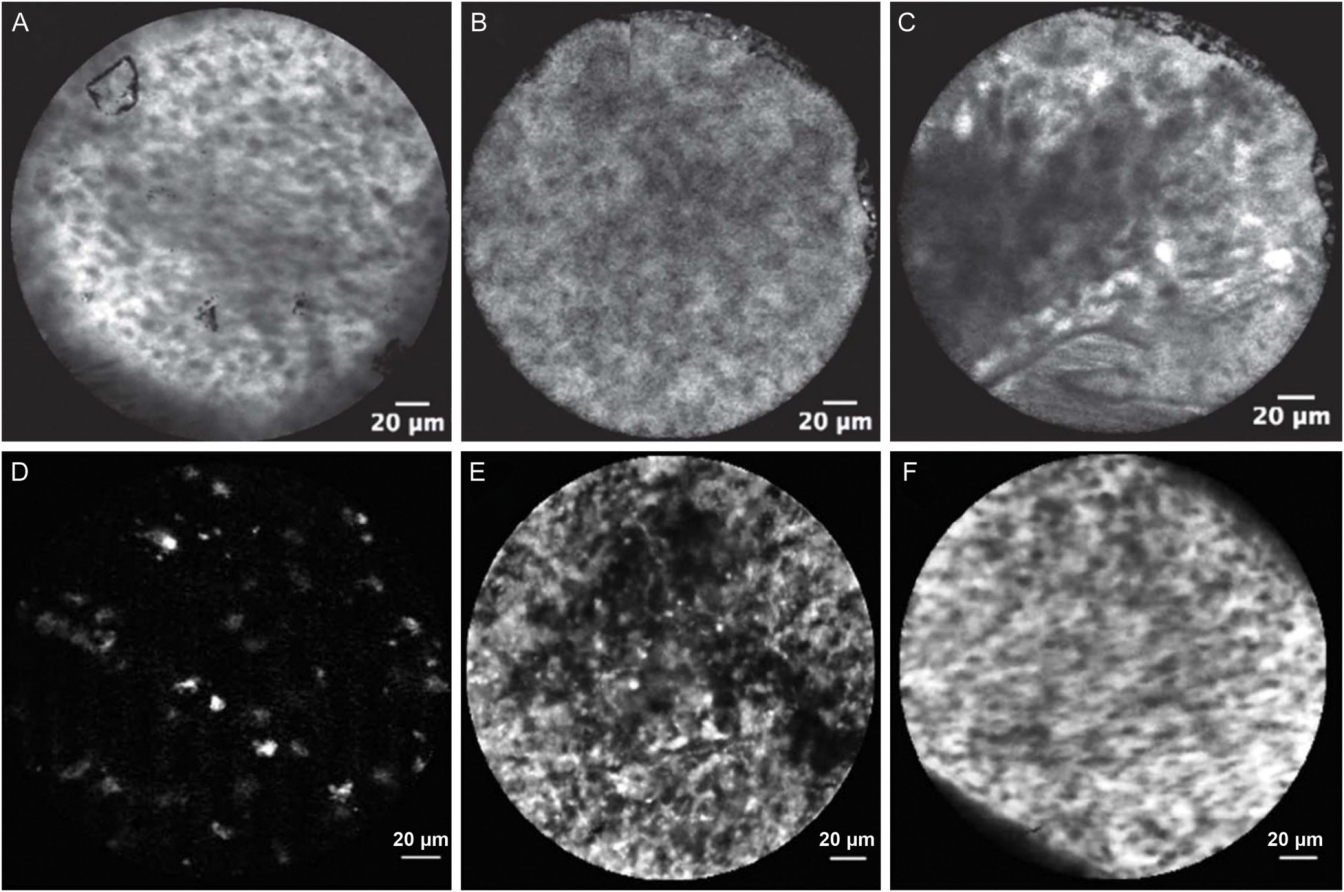
Examples of CLE images acquired *in vivo* using cellvizio with FNa in **(A)** grade 2 oligoastrocytoma, **(B)** grade 3 oligoastrocytoma and **(C)** glioblastoma, and with 5-ALA in **(D)** normal cortex, **(E,F)** glioblastoma. Adapted with permission from Pavlov et al. (2016) (22), © 2016 by the Congress of Neurological Surgeons.

There are a few other CLE systems that have been tested for neurosurgery use. Cellvizio is a CLE system featuring a collection of flexible miniprobes with different sizes, FOVs, resolution and working distances. It is primarily used with gastroenterological, pulmonological and urological endoscopes. Charalampaki et al. (21) reported the first experience of Cellvizio in neurosurgical procedures. Using a 2.5 mm GastroFlex probe, 150 fresh intracranial and intraspinal tumor samples were examined *ex vivo* with and without topical 0.01 mg/mL acriflavine that highlighted the cell nuclei. Across the 150 cases, CLE provided high-quality histomorphological images that closely mirrored traditional H&E sections. In another *in vivo* study of 9 presumed glioma patients, Pavlov et al. (22) used both FNa and 5-ALA as fluorescent contrast with Cellvizio (Figure 5). In 6 cases, CLE imaging was done with intravenous FNa. Vessel walls and individual blood cells were readily visualized without extravasation in normal tissue. While in tumor tissue, it was challenging to establish tumor grade due to limited visualization of mitoses, endothelial proliferation and necrosis. 5-ALA was given to 3 patients which produced discernible cytoplasmic and intercellular fluorescence in high-grade regions but was hampered by suboptimal excitation/emission overlap. The small dimension of the probe makes it especially advantageous in stereotactic biopsy and neuroendoscopy cases. Later, Charalampaki et al. (23) reported their experience of simultaneous widefield and confocal imaging of brain tumors in 13 patients using a multispectral fluorescence microscope and Cellvizio-780 nm CLE system. This system differed from the previous Cellvizio system in that it operated in the NIR spectrum. ICG was given intravenously at a dose of 50 mg 1 h before operative exposure of the tumor. CLE imaging was done *in vivo* in the resection bed and *ex vivo* in the tumor. ICG accumulated in the cytoplasm around the nuclei. In gliomas, CLE imaging managed to show ICG uptake at a subcellular level while widefield fluorescence imaging struggled to consistently detect fluorescence. In other tumor types, CLE showed characteristic histological features.

EndoMAG 1 is an autofluorescence-based CLE system that operates a 670 nm red laser. It has a round FOV with a diameter of 300 µm and a scanning speed of 40 images per second. The focal depth can be adjusted up to 80 µm (24). No fluorophore is given prior to imaging. Breuskin et al. (25) conducted a blinded comparison of *ex vivo* CLE imaging with EndoMAG 1 vs. frozen sections in 100 fresh neurosurgical specimens, reporting diagnostic sensitivities of 82%–90% for gliomas, schwannomas, and meningiomas and a lower 37% sensitivity for metastases. In the images, fluorescence was mostly seen in the background, with distinct patterns correlating to different pathologies, while the cells appeared as dark, round, polymorphous structures with a varying degree of density depending on tumor type and grade. The authors believed that diagnosis of brain metastases was particularly difficult due to their variable and heterogeneous morphology, making it harder to outline a common aspect. Radtke et al. (24) evaluated *ex vivo* autofluorescence CLE images of resected tumor tissue acquired from 36 patients with eight histomorphological criteria. Among these criteria, grey and white matter architecture, hypercellularity, necrosis, nuclear pleomorphism, hypervascularity and glial matrix change to be the most reliable, while mitotic activity was not identified. The presence of necrosis could be used to differentiate high- and low-grade gliomas.

## Discussion

This scoping review provides an overview of the current state of clinical application of various CLE imaging systems. Among these, CONVIVO and cCeLL have received both US FDA clearance for *in vivo* use (26, 27). CONVIVO is equipped with advanced imaging components with the highest resolution among all CLE systems, capable of generating very high quality images. However, this is achieved at the expense of low scanning speed. At maximum resolution, an image is generated every 1.3 s. When the probe is used handheld for *in vivo* imaging, unsteadiness of the probe and brain pulsation may cause motion artifacts that obscure part of or the whole image. This is improved with user experience but cannot be eliminated (14). The adjustable focal depth and built-in z-stack function support high quality imaging at a depth up to 30 µm beneath the surface of the imaged tissue (13).

### FNa and autofluorescence

CONVIVO is paired with intravenous FNa currently in clinical use. FNa crosses disrupted blood-brain barrier (BBB) in brain tumors and remains outside the cells with little to no cellular uptake by glioma and meningioma cells and to a varying degree by metastatic tumor cells (28, 29), creating images with bright backgrounds interspersed with dark cells. The administration protocol of the studies varied. In centers where widefield FNa fluorescence imaging is part of the standard of care, FNa was administered at the time of induction of anesthesia and potentially over an hour prior to CLE imaging. In other centers, FNa was given 20–40 min or only a few minutes before imaging. This variation may contribute to some extent to the difference in the results across studies, but previous analysis showed that the effect is not significant (30). It is well known that relatively preserved blood-brain barrier in non-enhancing brain tumors leads to low FNa concentration in these tumors, which in turn may affect the imaging quality and interpretation. Higher doses of FNa may increase image quality and reveal histological features that are otherwise not captured (11). However, higher dose FNa may not be possible due to protocols and regulations.

Detection of autofluorescence by CLE imaging had been largely overlooked due to the fact that it is frequently obscured by the fluorescence of FNa. Recent report by Reichenbach et al. demonstrated that patterns of autofluorescence are associated with different tissue types, especially nontumor brain tissues, glioblastomas, and metastatic brain tumor (16). Although label-free CLE imaging solely based on tissue autofluorescence has limited diagnostic reliability based on current studies (16, 24, 25), autofluorescence patterns may provide additional information to the interpretation of FNa-based CLE images.

### Near-infrared CLE

cCeLL uses a resonant scan method with Lissajous pattern rather than raster scanning and boasts a significantly higher image acquisition rate of up to 10 frames per second at a 500 × 500 µm FOV (20). This high scanning speed may reduce motion artifacts *in vivo*, thereby minimizing the number of uninterpretable images, although no reports of *in vivo* neurosurgical use have been published with cCeLL yet. Its NIR laser is designed for ICG, a fluorophore predominantly used for intraoperative widefield visualization of vasculature. ICG has been evaluated as an agent for fluorescence guidance in brain tumor surgery (31, 32), especially with the introduction of second-window ICG technique by Lee et al. (33, 34) Widefield detection of ICG fluorescence requires special camera systems since it is outside the visible light wavelength spectrum. On the other hand, NIR CLE systems show the fluorescence at the subcellular level, making them a powerful addition to this fluorescence technique. Early experimental work using a prototype NIR CLE system showed that ICG specifically accumulates in the cytoplasm, leading to high specificity of ICG to delineate tumor cell morphology (3), but such specific imaging equivalence has not been replicated in clinical NIR use for intraoperative neurosurgical CLE imaging.

### Diagnostic performance of CLE imaging

Although multiple studies report that CLE achieves diagnostic performance comparable to frozen section, these findings must be interpreted with caution. The variations in study design, mainly in the sampling methods, fluorophore dosage and timing, and outcome definitions, led to very different results. With the most studied CONVIVO, a study that only sampled tumor core reported accuracy exceeding 90% across all tumor types (17), while studies that specifically sampled glioma margins showed 75%–85% accuracy (1, 18). Furthermore, small sample size in these studies may lead to sampling bias and drastically different sensitivity and specificity values. Timing and dosing of fluorophore administration may further contribute to variability, as early or repeated fluorescein injections often improve contrast, whereas delayed imaging diminishes interpretability (35). These methodological and biological differences underscore that CLE does not perform at the same level across all contexts. Rather, its strongest performance is observed in well-demarcated tumor cores, with more limited reliability in infiltrative margins. A more rigorous standardization of imaging protocols and reporting metrics is needed to definitively determine the true diagnostic performance (1, 17, 18, 35).

### CLE detection of glioma margin

Retrospective analyses based on some of the data reported in the articles included in this review were conducted with pooled CLE images collected at different institutions. Two studies systematically examined the diagnostic performance of CONVIVO at glioma margins (36, 37). Four neuropathologists and four neurosurgeons retrospectively reviewed images from 56 optical biopsies acquired *in vivo* at glioma margins. Their interpretations were compared to the matched H&E-stained histology acquired at the same location. The results revealed moderate interrater reliability and low overall CLE-H&E concordance and specificity. At tumor margins, lower number of tumor cells and presence of reactive nonneoplastic cells likely complicate the images and their interpretation. These results highlighted the need for improvements in the imaging system, the fluorophore, and the interpretation guideline for reliable intraoperative glioma resection margin assessment. The ability of FNa CLE to reliably elucidate regions of treatment effect from tumor tissue is significant, and especially relevant in cases of recurrent gliomas.

Another study compared *in vivo* CONVIVO imaging and widefield 5-ALA fluorescence imaging at glioma margins (38). Simultaneous widefield 5-ALA fluorescence imaging, *in vivo* FNa CLE imaging, and tissue biopsy were done and collected from 88 samples at glioma margins. Comparing results of the two fluorescence imaging modalities to histology showed that widefield 5-ALA fluorescence had significantly higher specificity, it frequently underestimated tumor invasion that could be detected by CLE imaging. Despite their retrospective nature and limited sample size, these studies uncovered important information on the true performance of CLE imaging at glioma margins that was overlooked in the prospective studies.

### Efficiency, workflow, and AI with intraoperative CLE

Importantly, our current understanding of the real-life impact of CLE imaging remains limited. Analysis of CLE imaging time during clinical cases showed that it is associated with significant time efficiency compared to traditional frozen section (1, 39). Costs are comparable (using US Medicare rates) between CLE and frozen section, and lower than those for 5-ALA imaging (1). Studies included in this review are not designed to reveal the clinical benefits, but appear to reveal benefits for local surgical tissue interrogation. Observational in nature, these studies demonstrated the feasibility and potential of CLE, they do not address whether its intraoperative use translates into meaningful outcome benefits such as optimal extent of resection of tumor and improved overall survival. To bridge this gap, well designed interventional studies are needed in which CLE is used to actively guide surgical decision-making. Such studies would provide critical data on whether CLE is truly beneficial.

As with other medical imaging, the incorporation of AI models into the CLE imaging workflow is advancing rapidly. Early convolutional neural network (CNN) models with FNa CLE reliably distinguished diagnostic from nondiagnostic frames (40), while weakly supervised CNNs can localize diagnostically relevant subregions without pixel-level annotations (41). An improved explanation method, XRelevanceCAM, improved alignment between image regions related to AI model decision making and clinically important structures in CLE images (42). Deep learning approaches trained on *ex vivo* FNa CLE have achieved high accuracy in differentiating glioblastoma, meningioma, and metastases, with performance further improved by excluding low-confidence or artifact-laden frames (43). AI systems combined with FNa CLE may be of assistance in diagnosing difficult cases, such as distinguishing malignant characteristics of MRI-gadolinium-non-enhancing brain tumor CLE images or video loops (44). Style transformation of monochromatic FNa CLE images into colorized HE-stained-look-like images via AI processes shows unique potential to enhance and discriminate regions of the CLE image that are difficult to interpret (45).

Although most work to date on AI-assisted interpretation of neurosurgical CLE images was retrospective with prospective intraoperative validation lacking, outside neurosurgery, real-time AI–CLE pipelines have demonstrated ultrafast, sub-0.05 s frame processing and highly accurate differentiation of gastric cancer from normal mucosa, underscoring the potential of intraoperative deployment as a standalone tool or a supplement to human expert interpretation (46). Collectively, these systems vastly speed up filtering out of artifactual or noninformative images and evaluation of images that may be important for the diagnosis of aggressive tissue regions, thereby represent the promising future of fast, automated intraoperative histology assessment.

### Advantages, limitations, and improvements for CLE

The limited FOV of CLE systems described in this review prohibits histological diagnosis and subtyping in most instances unless pathognomonic structures are visualized. Therefore, the CLE systems seem to be more suitable as a surgical tool to inform the surgeon, with pathologists’ input, whether there is tumor or lesional tissue present or not. This is especially relevant in the marginal regions of a glioma resection bed, near or within functionally eloquent brain regions, or when the surgeon is operating in an area of recurrent or residual tumor vs. posttreatment brain effect. CLE may have advantages as well in the midst of the tumor or mass to rapidly assess histological features. This would seem particularly relevant for immediate real-time imaging of gadolinium non-enhancing masses that are often suspected to be of lower grade but that, in fact, turn out to be aggressive. The ability to interrogate low-grade gliomas or non-enhancing gliomas or other masses presents an advantage, as a digital optical biopsy can be rapidly assessed within seconds with confidence to guide the surgeon's next operative maneuvers. The CONVIVO FNa-based system has achieved claims in Europe that it can discriminate intraoperatively between tumor and non-tumor tissues, although the FDA has not approved similar claims for use in the US neurosurgery marketplace.

At present, only two main fluorophores (FNa and ICG) are practical and approved by the FDA and in Europe with CLE systems, although a third fluorophore (5-ALA) has been explored with a non-clinical system (44). Development or use of more specific-acting fluorophores within the blue laser range for CLE would significantly aid the lack of specificity, as shown by FNa CLE imaging. Such fluorophores as acriflavine, acridine orange, and cresyl violet show high affinity for intracellular structures, especially it seems for tumor cells (47). Although these fluorescent dyes can be applied topically in gastrointestinal endoscopic procedures to identify precancerous or cancerous sites for biopsy, the FDA has not allowed them to be used in the brain due to their imputed mutagenic effect. Potentially, the small molecule size of FNa could be leveraged and conjugated with specific probes for tumors, especially for use in identifying glioma cells. Initial work with ICG used with a NIR system showed an apparent advantage for such an increase in specificity for tumor tissue identification (3). A scanning fiber endoscope system similar to the CLE systems referenced here and based on detection of protoporphyrin IX showed the clear margin area of 5-ALA-induced fluorescence in an experimental murine malignant glioma, however the system was not developed for clinical use (48).

However, studies investigating the capability of the images to reveal benign and malignant characteristics have been limited and require expansion. Although the systems can detect histoarchitecture and give impressions of what an individual cell may be, there are no studies that correlate what a selected single cell within the image frame may actually be, and such becomes more problematic at the margin as studies have shown. For example, distinguishing between tumor cell, reactive astrocyte, inflammatory cell, and even red blood cells (especially with the FNa-based systems) can be problematic. Image interpretations require a learning curve, contextual clues of other cells in the frame, and relevant clinical information.

The use of CLE systems has largely focused on primary invasive brain tumors, and to a much lesser extent on tumors such as metastases, meningiomas, neuromas, schwannomas, and even other head and neck, aural, and orbital tumors. Within these realms CLE may find special utilization such as exploration and interrogation for remnant tumor associated to sensitive structures, achieving clearly resected or tumor-free margins, discriminating nerve fibers within or complexed with tumor tissue, etc. head and neck, neuro-otological and otolaryngological surgeons may find use of CLE systems for optimal or improved surgical results.

Thus, the development of specific-acting fluorophores is of significant advantage. The hurdle toward use of the topical fluorophores mentioned or others with more specific actions is high in that the FDA considers them drugs and they must be investigated on their own and in combination with the imaging apparatus. Thus, approvals either require reconsideration by the FDA or large multicenter studies. At this point, the working interpretation when using CLE systems either in the mass of the tumor, or more realistically and practically, or at the border is, “Is it tumor or not?”

### Limitations of the scoping review process

This scoping review synthesized currently available clinical reports on intraoperative CLE imaging but is subject to several inherent methodological limitations. Because we aimed to review the breadth of available evidence, which is relatively scarce due to the novelty of this technology, we did not perform risk of bias assessments. The substantial heterogeneity in study design, imaging platforms, fluorophore administration protocols, sampling strategies, and outcome definitions may introduce bias into individual diagnostic performance estimates, and precludes formal meta-analysis and pooled diagnostic accuracy calculations. In addition, the predominance of single-center studies, many conducted by groups with extensive CLE experience, may limit generalizability. Considering that this technology is relatively new and not yet widely adopted, the reports are from a small group of users and the studies are mainly observational rather than interventional. So, risk of bias is inevitable and a proper assessment is not likely to provide value for our purpose.

Publicly available information on the CLE systems is not consistent, and the comprehensiveness of the reports on these systems varies significantly. Among these, CONVIVO and its predecessor, FIVE1, are the most studied and published. Cellvizio is not approved marketed for neurosurgery use and only descriptive and exploratory reported were published. EndoMAG 1 is a CLE system whose information beyond the reports included in this review is not available. cCeLL is a newly developed and approved system for which technical description and structured clinical studies have not yet been published. For the same reason, evaluating their performance relative to other platforms operating at similar conditions is also not possible, as the operating conditions of these systems are drastically different.

These limitations underscore the need for standardized study design and imaging protocol, studies with CLE linked to or associated with interventional neurosurgical procedures, and multi-center prospective studies to define the true clinical utility of intraoperative CLE imaging in neurosurgical practice.

## Conclusions

With over a decade of research, CLE has progressed from an experimental technology to an intraoperative imaging modality in neurosurgery, capable of delivering real-time, high-resolution visualization of brain tumor histoarchitecture. Across multiple CLE platforms, clinical reports consistently demonstrate feasibility, safety, and good diagnostic performance when used to image the core of the tumor, demonstrating the potential for integration into fluorescence-guided surgeries as a surgical guidance tool. However, current evidence is largely observational and focused on diagnostic accuracy, leaving its impact on key surgical endpoints, such as extent of resection and patient survival uncertain. The incorporation of AI into CLE interpretation offers powerful solutions for rapid frame triage, tumor classification, and explainable feature localization, further enhancing its intraoperative utility.

To fully realize CLE's potential as an optical biopsy tool, future research should prioritize well-designed interventional trials that evaluate CLE guidance on surgical decision-making and long-term outcomes, alongside continued optimization of imaging systems, fluorophores, correlation to tumor histopathological subtypes, and AI-assisted image analysis.

## Data Availability

The original contributions presented in the study are included in the article/Supplementary Material, further inquiries can be directed to the corresponding author.
