# Supplementary material for: Clinical application of confocal laser endomicroscopy in neurosurgery: a scoping review
Source: Front Surg. 2026 Jan 28;12:1715836. doi: 10.3389/fsurg.2025.1715836 (PMC12892495; doi:10.3389/fsurg.2025.1715836)
Supplement: Supplementary file 1 [file Table1.docx]

**SUPPLEMENTAL MATERIALS**

**Supplemental Table 1.** Search strategy for PubMed, Scopus and Embase. All searches conducted on June 20, 2025

| **Database** | **Query** | **Results** |
| --- | --- | --- |
| PubMed | ("Confocal"[Title/Abstract] OR "endomicroscopy"[Title/Abstract] OR "confocal endomicroscopy"[Title/Abstract] OR "confocal laser endomicroscopy"[Title/Abstract] OR "confocal imaging"[Title/Abstract]) AND ("glioma"[Title/Abstract] OR "brain tumor"[Title/Abstract] OR "neurosurgery"[Title/Abstract]) | 111 |
| Scopus | TITLE-ABS("Confocal" OR "endomicroscopy" OR "confocal endomicroscopy" OR "confocal laser endomicroscopy" OR "confocal imaging") AND TITLE-ABS("glioma" OR "brain tumor" OR "neurosurgery") | 116 |
| Embase | ("confocal" OR "endomicroscopy" OR "confocal endomicroscopy" OR "confocal laser endomicroscopy" OR "confocal imaging").ti,ab. AND ("glioma" OR "brain tumor" OR "neurosurgery").ti,ab. | 152 |
